# Expression of Behavioural Traits in Goldendoodles and Labradoodles

**DOI:** 10.3390/ani9121162

**Published:** 2019-12-17

**Authors:** Victoria L. Shouldice, A. Michelle Edwards, James A. Serpell, Lee Niel, J. Andrew B. Robinson

**Affiliations:** 1 Center for Genetic Improvement of Livestock, Department of Animal Biosciences, University of Guelph, 50 Stone Road East, Guelph, ON N1G 2W1, Canada; andyr@uoguelph.ca; 2 Ontario Agricultural College, University of Guelph, 50 Stone Road East, Guelph, ON N1G 2W1, Canada; edwardsm@uoguelph.ca; 3 Department Clinical Sciences and Advanced Medicine, School of Veterinary Medicine, University of Pennsylvania, 3800 Spruce Street, Philadelphia, PA 19104, USA; serpell@vet.upenn.edu; 4 Department of Population Medicine, University of Guelph, 50 Stone Road East, Guelph, ON N1G 2W1, Canada; niell@uoguelph.ca

**Keywords:** genetics, cross-breeding, dogs, behaviour, IGF1, doodles, crossbred, hybrid, C-BARQ

## Abstract

**Simple Summary:**

Crossbred dogs are gaining in popularity with the general public, but we do not fully understand how these crossbreds behave compared to their parent breeds in regard to inherited behaviour traits. Because of this, we investigated behaviours exhibited by crossbred dogs by focusing on the popular Goldendoodle and Labradoodle crossbreds by comparing them to their corresponding parent or constituent breeds: Standard or Miniature Poodle, and Golden Retriever or Labrador Retriever. The data for this study was provided by 5141 volunteer dog owners from across the world who filled out the Canine Behavioural Assessment and Research Questionnaire (C-BARQ) online survey. The survey results were used to analyse fourteen different representative behavioural trait scores. As expected from a first-generation crossbred (F1), the crossbreds in our study tend to fall between the two parent breeds with some exceptions. The Goldendoodle displayed more problematic behaviour when compared to its constituent breeds, whereas the Labradoodle only differs significantly from the Miniature Poodle in dog rivalry. These results can help advise future dog owners on behavioural trends for particular crossbreds.

**Abstract:**

As crossbred dogs gain in popularity, how they express inherited behaviour traits in comparison to their purebred constituent breeds is of interest. We investigated behaviours exhibited by crossbred dogs by focusing on the popular Goldendoodle and Labradoodle crossbreds and comparing them to their corresponding constituent breeds: Standard and Miniature Poodle, Golden Retriever or Labrador Retriever. The data for this study was provided by 5141 volunteer dog owners who filled out the Canine Behavioural Assessment and Research Questionnaire (C-BARQ) online survey. The survey results were used to analyse breed differences in fourteen representative behavioural trait scores: trainability, stranger-directed aggression, owner-directed aggression, dog-directed aggression, dog rivalry, dog-directed fear, stranger-directed fear, non-social fear, touch sensitivity, separation-related problems, excitability, attachment/attention-seeking behaviours, energy and chasing. As expected from a first-generation crossbred (F1), the crossbreds in our study tend to fall between the two constituent parent breeds with some exceptions. Our results suggest that the F1 Labradoodle differed significantly from one of the pure constituent breeds only in dog rivalry, whereas the F1 Goldendoodle behaviour varied from one or more pure constituent breeds in dog rivalry, dog-directed aggression, dog-directed fear, and stranger-directed fear. These results can help advise future dog owners on behavioural trends for particular crossbreds.

## 1. Introduction

It is estimated that there are currently over 400 different breeds of dogs around the world [1]. A breed is a closed population of closely related animals which generally results in individuals with very similar physical attributes, relatively predictable behaviour, and in some cases the selected ability to perform predetermined jobs [1,2,3,4]. In addition, the dog-owning public is moving beyond the long-standing pure-breeds and embracing crossbred dogs, with databases of dog breeds suggesting that crossbred dogs make up one-third of the world’s dog population and that this portion of the population is growing [5,6,7].

It is not uncommon to encounter a purpose-bred crossbred dog nowadays as people are drawn more and more to designer dog breeds known as Doodles [8]. Doodles are a cross between a popular breed such as a Labrador Retriever or Golden Retriever and a Poodle [8]. This is done with the intent of producing a non-shedding hypoallergenic dog that is similar to the non-Poodle parent [8]. Doodles are not new; the idea of this group of crosses is originally credited to an individual named Wally Conran. Wally worked for the Royal Guide Dog Association of Australia in the 1980s and was trying to create a guide dog that was also hypoallergenic [8]. To do this, he crossed his best breeding Labrador with a Poodle; much to Wally’s surprise he ran into an issue with people not wanting to foster the crossbred puppies [8]. To solve this, Wally came up with the name Labradoodle and marketed this cross as a new hybrid dog breed [8]. The marketing worked as the public gravitated to this idea of having a well-loved dog breed that was hypoallergenic without shedding [8]. Unfortunately, Wally soon came to realise that since these dogs were hybrids, they did not have the same predictable temperament or morphological aspects that purebreds have [8]. Even so, the public has taken to these crosses and their popularity has grown since [8]. Doodles have followed public demand and trends in dog ownership with more and more crosses being developed, such as the Cockapoo (Cocker Spaniel, Poodle cross), Bernadoodle (Bernese Mountain Dog, Poodle cross), and Shepadoodle (German Shepherd, Poodle cross) to name a few [8].

It is generally believed that crossbreds are healthier, have better temperaments, and are better all-around dogs due to the absence of inbreeding depression [5,9,10,11]. Purebred dogs are known to differ between breeds in general temperament and personality traits, and although there have been many studies [1,4,5,6,12] of these inherited behaviours in purebred dogs, there is still much to be understood about the specific links between behaviour and genetics [4,5,12]. Crossbreeding blends DNA from different parent breeds, and while the interaction and expression of alleles is not fully understood [3,4,9,11,13], genetic theory would suggest that crossbreds should express behaviours intermediate to those of the constituent breeds [14]. While crossbreeding is thought to be useful for combining favourable characteristics from two breeds, little is known about how temperament and personality changes when crossing two purebred dogs, nor how the expressed behaviour of progeny relates to that of the parent breeds [4,5,15]. Behaviour expression in canines is not measured on a binary scale where a dog either expresses or does not express the behaviour in question [4]. Instead, behaviour is measured on a relative scale of expression and dogs, even in purebreds, will express a set behaviour to different degrees [4]. It should be noted that some purpose-bred behaviours, such as herding and pointing, will only be present in breeds that have been selected to point or herd. However, most non-specialized dog behaviours (e.g., fear, aggression, trainability, excitability) are measured on a spectrum and show variability between breeds [1]. Due to the nature of behaviour quantification and our understanding of quantitative genetics [14], where the parent breeds differ in score for a particular trait, it is expected that crossbred dogs will express an intermediate level of the behaviour [4]. We define here an intermediate level of behaviour expression as one that falls between those of the constituent parent breeds. For traits that are scored on a scale of expression, that intermediate expression would be expected to be the average of the scores of the parent breeds. In the cases where the constituent parent breeds do not differ significantly from one another in average behaviour trait expression score, we would expect the crossbreds to be consistent with that average expression score of the parent breeds as well. With the increase in popularity of crossbreeds, it is important to fill the gaps in current knowledge to improve our understanding of the ways in which crossbred dog temperament and personality compare to those of their constituent breeds.

Our objective was to investigate behaviours exhibited in crossbreds and compare the expression of those behaviours to the expression of the same behaviours by the constituent pure-breeds. It is widely believed that an F1 hybrid should inherit predictable behaviour as first described by Darwin [16]. We focused on Labradoodle and Goldendoodle, which result from crossing a Standard Poodle or a Miniature Poodle with either a Labrador Retriever or a Golden Retriever. As all of the parent breeds, including the poodles, were used for hunting game birds, it is thought that these dog breeds would be more similar to one another than to non-retriever breeds and crosses, such as the Shepadoodle. Because the Miniature Poodle and Standard Poodle are two different breeds as defined by the Canadian kennel club, and since the Canine Behavioural Assessment and Research Questionnaire (C-BARQ) database does not ask owners for pedigree information, it was necessary to compare both Poodle types to the Doodle crosses. Thus, we predicted that the Labradoodle and Goldendoodle, having parent breeds from similar working backgrounds, would be similar to their parent breeds with little behavioural tendency towards one or the other parent breed [15].

To test this prediction, we are utilizing the Canine Behavioural Assessment & Research Questionnaire, which is a well-validated questionnaire-based research tool that has been used extensively in peer-reviewed studies on dog behaviour [17]. C-BARQ is an owner-completed questionnaire originally developed by Yuying Hsu and James Serpell [18], that assesses 14 different behavioural traits based on questions reflecting the responses of dogs to various real-life scenarios. Data collection has been ongoing since 2005, and the current database has over 50,000 dog behavioural records from around the world and includes over 300 different crossbreds and purebred dogs [17,18]. The database was originally created to be used for assessing potential problematic behaviour within the working and pet dog communities [18]. Because of the large database, we are able to collect phenotypic behavioural profiles which allow us to assess behavioural profiles of the purebreds and Doodles in our study even in the absence of pedigree information.

## 2. Materials and Methods

### 2.1. Data Collection

The data for this study were generated from volunteer dog owners who filled out the C-BARQ online survey hosted by the School of Veterinary Medicine, University of Pennsylvania (https://vetapps.vet.upenn.edu/cbarq/). The C-BARQ project was launched in 2005 and survey data have accumulated since that time. Data for this study were extracted and cleaned in April 2019. Data were cleaned by removing identifiers of owners such as emails, non-complete C-BARQ surveys, and dogs with health issues such as hypothyroid, hyperthyroid, Addison’s, severe allergies, or any dogs with a disease that requires medications that may alter behaviour. Dog owners are able to complete the C-BARQ survey online and were originally made aware of the survey via veterinary offices, dog trainers, social media, pet magazines and word of mouth [17]. The survey is organised by owner ID and each volunteer can complete the survey for up to a maximum of 10 dogs. Owner effect is a concern in the current dataset due to the relatively small number of crossbreds, as multi-dog households could presumably have similar behaviours due to environment [18]. We have addressed these concerns in our statistical model. Due to the nature of the C-BARQ project, we do not have pedigree information but do have information on where the dog was acquired. We also had no control over who filled out the survey.

### 2.2. Behavioural Measures

Fourteen different representative behavioural traits were previously extracted by factor analysis and were included in this study: trainability, stranger-directed aggression, owner-directed aggression, dog-directed aggression, dog rivalry, dog-directed fear, stranger-directed fear, non-social fear, touch sensitivity, separation-related problems, excitability, attachment/attention-seeking behaviours, energy and chasing. These scores were determined by owners rating their dogs’ behaviour in 100 different scenarios (e.g., when approached directly by an unfamiliar adult while being walked/exercised on a leash) [18]. Each question was scored on a scale from 0–4 to denote the frequency (0 = never; 1 = seldom; 2 = sometimes; 3 = usually; 4 = always), or severity (0 = no signs of the behaviour; 1–3 = mild to moderate signs of the behaviour; 4 = severe signs of the behaviour) [18]. From there, questions were organised into eight categories, three of which are based on severity and five based on the frequency of occurrence [18]. For each factor, scores for the related questions were combined to produce an average score that was used for analysis.

### 2.3. Statistical Analyses

Separate analyses were completed for Labradoodle and Goldendoodle crosses. Thus, the two analyses included (1) Miniature Poodle, Standard Poodle, Labrador Retriever and Labradoodle **(LD)**, and (2) Miniature Poodle, Standard Poodle, Golden Retriever, and Goldendoodle **(GD)**. There were a total of 5141 dogs in both data sets with 166 Labradoodles, 2597 Labrador Retrievers, 157 Goldendoodles, 1366 Golden Retrievers, 597 Standard Poodles and 258 Miniature Poodles making 3618 dogs in the LD analysis and 2378 dogs in the GD analysis. Individual dogs were assumed to be unrelated and we do not know which Poodle type was used for each of the crossbred dogs.

All statistical analyses were performed using SAS version 9.4; analyses were considered significant if *p* ≤ 0.05 [19]. Outcome variables were examined using mixed linear regression models using the GLIMMIX procedure. Comparisons were assessed using adjusted least-square means and adjusted (using Tukey Kramer adjustment) to account for the uneven sample size between our breeds. The country was included in the models as a fixed effect. The single trait linear animal model used in the analysis was:

Y_ijklmnop_ = µ_i_ + breed_j_ + sex_k_ + country_l_ + where acquired_m_ + ageatevaluation_n_ + owner_o_ + a_p_ + e_ijklmnop_

where,

⚬ y_ijklmn_ is the behavioural observation for animal o;
⚬ µ is the overall mean for the observation on trait i;
⚬ breed (j) was a fixed effect (levels are equal to Labradoodle, Labrador Retriever, Goldendoodle, Golden Retriever, Miniature Poodle and Standard Poodle);
⚬ sex is a fixed effect (k, being either female or male);
⚬ country of residence of the owner completing the survey is a fixed effect representing the environment and culture the dog is from;
⚬ where aquired is a fixed effect of where an owner acquired their dog (m, being either a breeder, pet store, bred by owner, shelter, friend or relative, stray or other);
⚬ age at evaluation is the fixed effect of the age of the animal when it was evaluated in the C-BARQ system (n, is the category of the dogs’ age; puppy, junior, adult, senior);
⚬ owner is the random effect of o on the household interaction;
⚬ a is the random additive genetic effect of animal p;
⚬ the assumptions for the random effects include: $e\;\sim \;N\left(0,{I\sigma }_{e\;}^{2}\right)$ $\sigma_{e\;}^{2}$ is the residual variance, and I is an identity matrix.

For all models, residuals were tested for homogeneity and normality by using the Shapiro-Wilk test and plots. Due to a lack of parentage information, in a separate analysis, we attempted to discern the contributing breed of Poodle in the Labradoodle and Goldendoodle crossbreds by analysing the weight of the crossbreds relative to the weight of the purebreds using the “*Proc univariate*” function in SAS for all breeds in the LD and GD analysis.

## 3. Results

### 3.1. Labradoodle

**Figure 1:**
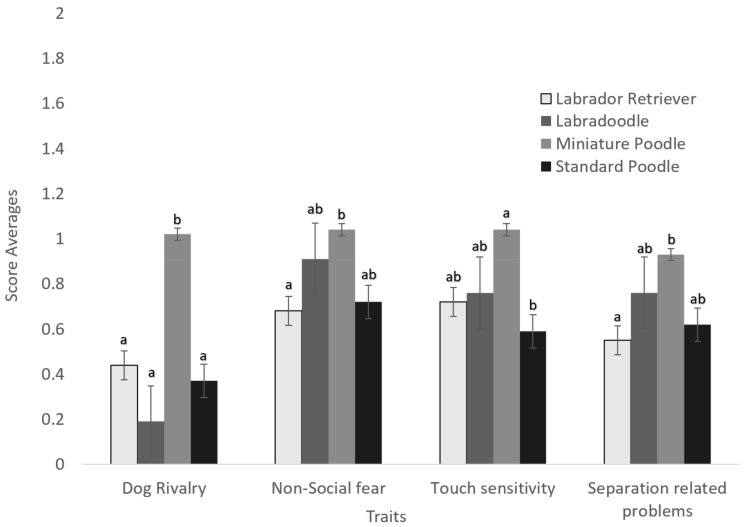
Breed average behavioural scores collected from C-BARQ for the Labrador Retrievers, Labradoodles, Standard Poodles and Miniature Poodles. The traits displayed above were found to have significant differences (*p* < 0.05) between the breeds in the analyses. Significant differences between breeds are marked with a and b. ab indicates that this breed did not vary significantly from either breed a or breed b. Behavioural scores are ranked from 0–4, zero being never and 4 being always. Error bars on the graph are standard error.

For the LD analysis, four behavioural scores differed between breeds (Table 1, Figure 1). Labrador Retriever and Standard Poodle scores did not differ significantly from those of Labradoodles for any behaviour category. In contrast, Miniature Poodles scored significantly higher than Labradoodles for dog rivalry. The Miniature Poodle had a higher score than the Labrador Retriever in both non-social fear and separation-related problems, whereas the Standard Poodle had a lower score than the Miniature Poodle for touch sensitivity. There were a number of interactions between Breed and other fixed effects. The interaction with Where Acquired was significant for all of the models, and interactions with Sex, Country, and Age at Evaluation were significant for most of the models.

### 3.2. Goldendoodle

**Figure 2:**
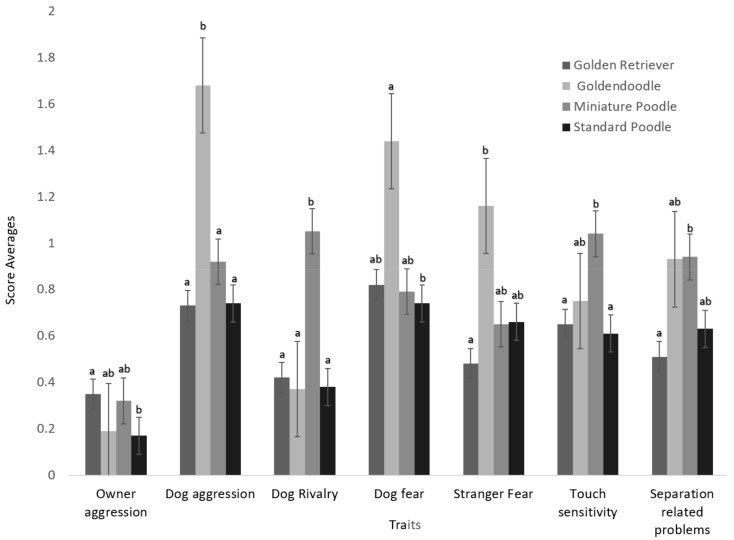
Breed average behavioural scores collected from C-BARQ for Golden Retrievers, Goldendoodles, Standard Poodles and Miniature Poodles. The traits displayed above were found to have significant differences (*p* < 0.05) between the breeds. Significant differences between breeds are marked with a and b. ab indicated that this breed did not vary significantly from either breed a or breed b. Behavioural scores are ranked from 0–4, zero being never and 4 being always. Error bars on the graph are standard error.

The GD analysis had seven behavioural scores that differed between the breeds (Table 2, Figure 2). The Standard Poodle had a lower score than the Golden Retriever for owner-directed aggression. The Goldendoodle differed from the Golden Retriever, Standard Poodle and Miniature Poodle in dog-directed aggression with a higher score than the other three breeds. The Miniature Poodle had the highest average score for dog rivalry out of all four breeds in the GD. The Goldendoodle had the highest average score for dog-directed fear and was significantly different than the Standard Poodle which had the lowest average score for this trait out of all the breeds in this analysis. The Golden Retriever scored significantly lower for stranger-directed fear on average when compared to the Goldendoodle. On the other hand, the Goldendoodle did not differ significantly from the Miniature Poodle for average scores for owner-directed aggression, dog-directed fear, stranger-directed fear, touch sensitivity, and separation-related problems. The Miniature Poodle differed from the Golden Retriever and Standard Poodle with higher average scores for both dog rivalry, and touch sensitivity.

Due to the lack of pedigree information in the current study, we examined the distribution of dog weight for both the LD and GD analyses to determine if we could predict Miniature or Standard Poodle as the poodle parent breed of the Goldendoodles or Labradoodles. The weights of the Goldendoodle and Labradoodle were evenly distributed between the Standard and Miniature Poodle so differentiation of the poodle breed in the crossbreds was not possible.

## 4. Discussion

As the Labradoodle and Goldendoodle are expected to be F1 crosses, one would expect these crossbreds to demonstrate behavioural score phenotypes that are intermediate to their purebred parent breeds. This was generally true in this study with some notable exceptions. We found that the Labradoodle differed from the Miniature Poodle for average score in only one trait, dog rivalry, and the Goldendoodle differed from various different parent breeds for average score in four traits—dog-directed aggression, dog-directed fear, stranger-directed fear, and dog rivalry.

There were some similarities across the LD and GD analyses; for instance, the Miniature Poodle had the highest average score for dog rivalry for both the LD and GD analyses. In this study, dog rivalry was defined as “dog shows aggressive or threatening responses to other familiar dogs in the same household” [20]. However, rivalry does occur more frequently with Miniature Poodles than in the other pure- and cross-breeds, whose average scores are close to “almost never” from both LD and GD analyses.

Differences in separation-related problems were noted in both the LD and the GD analyses as well, with separation-related problems defined as “Dog vocalizes and/or is destructive when separated from the owner, often accompanied or preceded by behavioural and autonomic signs of anxiety including restlessness, loss of appetite, trembling and excessive salivation.” [18]. In both the LD and GD analyses, the Miniature Poodle had the highest average score for separation-related problems, and was significantly higher than the parent retriever breeds, with the crossbreds showing intermediate average scores that did not differ significantly from either parent stock. Dogs with higher levels of separation-related problems have been known to cause self-harm due to escape attempts [21]. Not only do separation-related problems have a negative impact on a dog’s welfare, but they also negatively impact the human-animal bond and in some cases lead to rehoming of the animal [21]. Further research is needed to determine whether Doodles resulting from Miniature Poodle crosses are more prone to this behaviour problem than those resulting from Standard Poodle crosses.

The last trait common to both the LD and GD analysis is touch sensitivity. This as defined as “Dog shows fearful or wary responses to potentially painful or uncomfortable procedures, including bathing, grooming, nail-clipping and veterinary examinations”. As with the other similar traits, the Miniature Poodle differed the most from the breeds with an average score of 1.04. In the GD analysis, the Miniature Poodle differed from both the Golden Retriever and the Standard Poodle, but in the LD analysis, only the Standard Poodle differed significantly from the Miniature Poodle.

Non-social fear is the only trait that showed significant differences in the LD analysis that was not also found in the GD analysis. Non-social fear is defined as “Dog shows fearful or wary responses to sudden or loud noises (e.g., thunder, traffic and unfamiliar objects and situations).” The Miniature Poodle had the highest average score in this analysis and scored significantly higher than the Labrador Retriever. Labradoodles had an intermediate average score but did not differ from any of the parent breeds. These scores show that the Miniature Poodle displays some noise and object aversion, while the Labrador Retriever rarely shows such aversion. This may suggest that the Miniature Poodle has been directly or indirectly selected to be further removed from its origins as a hunting dog than the other purebreds. This trait, like separation-related problems, may also have an impact on the dog’s welfare, and there is also evidence that these two behavioural issues have an impact on each other, as the dog is already in an anxious state when the owner leaves [21].

The Goldendoodle had the highest average dog-directed aggression score in the GD analysis. Dog-directed aggression was defined as “Dog shows threatening or aggressive responses when approached directly by unfamiliar dogs” [18]. This was the only trait in the GD analysis where the Goldendoodle differed significantly from its constituent parent breeds. The other behaviour noted in the GD analysis was Owner-directed aggression which was defined as “Dog shows threatening or aggressive responses to the owner or other members of the household when challenged, manhandled, stared at, stepped over, or when approached while in possession of food or objects.” [18]. The Golden Retriever had higher average scores for owner-directed aggression than the Goldendoodle, Standard Poodle, and Miniature Poodle.

The Goldendoodle showed a higher average score for stranger-directed fear in the GD analysis compared to the purebreds, that was not observed between the Labradoodle and purebreds in the LD analysis. Stranger-directed fear is defined as “Dog shows fearful or wary responses when approached directly by a stranger” [18]. For most traits, the Goldendoodle was intermediate to the parent breeds, but in the case of stranger-directed fear, the average score for the Goldendoodle was higher than the Golden Retriever. The last behaviour that stood out in the GD analysis was dog-directed fear, as the Goldendoodle differed significantly from the Standard Poodle. The Goldendoodle had the highest average score for dog-directed fear; compared to the Golden Retriever, Standard Poodle and Miniature Poodle which all had much lower scores. Dog-directed fear was defined as “Dog shows fearful or wary responses when approached directly by unfamiliar dogs.” [18].

Based on our analyses of the purebred animals in this study, the Miniature Poodle differed the most from the other pure-breeds, including the Standard Poodle. We also observed the Goldendoodle differentiating from one or both parent breeds. The Goldendoodle differed significantly from a parent breed in dog-directed aggression, dog-directed fear, and stranger-directed fear. These differences may be linked to hybrid vigour [1,22,23]. Hybrid vigour is one of the reasons many of the pet-owning public acquire a Doodle-cross; crossbreds are perceived to be a healthier animal since hybrid vigour has a proven effect on fitness traits in other species such as livestock [22]. However, we do not fully understand the effect of hybrid vigour in the domesticated dog, nor how hybrid vigour affects behaviour [22]. An inbreeding study conducted by Mellanby et al. [23] looked at 25 different breeds, including the Golden Retriever and Labrador Retriever. The authors found that the Golden Retriever was one of the most inbred breeds examined in their study. As a result, the Goldendoodle would be expected to have a greater effect of hybrid vigour in comparison to the Labradoodle [22,23] and thus, potentially greater differences in behaviour from the constituent breeds than the Labradoodle.

The differences we are observing in the Miniature Poodle may be due to the contribution of the expression of the *insulin-like Growth Factor-1 (IGF1)* gene which affects the growth and stature of the dog. *IGF1* is expressed in many toy or miniature breeds but is rare in large breeds [1,24,25]. Interestingly, studies have found that there are significantly lower levels of circulating *IGF1* in the serum of Miniature Poodles in comparison to the Standard Poodle [1,26,27,28]. Some evidence shows that not only is this gene associated with growth, but it is believed to be linked to temperament as well [1,24,29]. A study conducted by Uhde and colleagues [29] bred German Shorthaired Pointers for fearful or nervous behaviour, and they found an inverse linear correlation between fearful behaviour and the expression of *IGF1*. This suggests that lower levels of *IGF1* expression make smaller dogs more fearful, reactive, excitable, and sensitive to touch [1,24,29]. Without pedigree information, we unfortunately, cannot know if there is any difference in behaviours between Doodles crossed with a Miniature Poodle or Standard Poodle; however, our results suggest there may be some differential expression of *IGF1* occurring. For instance, we observed that the Miniature Poodle differed from the other purebred dogs in dog rivalry, non-social fear, touch sensitivity, separation-related problems, dog-directed aggression, stranger-directed fear, and dog-directed fear, which are all behaviours considered to be higher among small breeds and could be related to expression of *IGF1* [1,24,29]. These behaviours that may be associated with *IGF1* expression are the same behaviours in our study that we saw significant average score differences between the LD and GD analyses. If the *IGF1* gene does influence fear and aggression, it could create welfare implications for the dogs with this gene expression. Fear is a negative affective state that can impair animal welfare, particularly when it occurs at high levels and is protracted [12,21,27]. It can also lead to aggression and other behaviour problems that result in a breakdown of the human-animal bond and increase the likelihood of euthanasia or relinquishment [12,21]. Based on our scores for dog-directed aggression and dog-directed fear behaviours in the Goldendoodle, it would appear this cross expresses elevated levels of aggression and fear-based behaviours, and that these issues are not present in the Labradoodle. Although the average scores observed in the current study are relatively low overall, it is important that owners are aware of potential impacts on dog behaviour and welfare [12,21,27].

Previous research has found that the Labrador and Golden Retrievers have comparatively low occurrences of aggressive behaviours [17]. In contrast to these predictions about one of their constituent parent breeds, we observed elevated levels of dog-directed aggression from the Goldendoodle, and we also saw higher levels of dog-directed fear and stranger-directed fear. This corresponds to what other studies have found when examining *IGF1* expression and crossbred dogs [7,26,28,29]. Bennett and Rholf [7] examined crossbred dogs and found them to be more fearful, aggressive, vocal and neurotic when compared to purebred dogs. Temesi and colleagues’ [30] study also agreed with Bennett and Rholfs’ [7] findings as they too noted neuroticism, dog-directed fear, and human-directed fear in cross-breeds. Similarly, Hsu and Sun [31] found that mixed breed dogs, in general, seem to have a higher expression of aggression and fear-based behaviours. This difference in crossbreds could be attributable in part to heterosis, although these studies did not differentiate between F1 hybrids such as the Goldendoodle and Labradoodle and mixed breeds of unknown origins [4,22,23]. Retained heterosis may be lower in mixed multi-breed dogs compared to heterosis in F1 crosses [4]

While the influence of the *IGF1* gene is one potential explanation for the behavioural differences observed in Miniature Poodles, behaviour in these dogs might also have been influenced by differences in the ways that small and large dogs are treated by owners. Arhant and colleagues [32] found that the size of a dog has an impact on how dog owners interact with the animal and that smaller dogs were more likely to have unfavourable behaviours, such as disobedience, increased aggression, excitability and fearful or anxious behaviours [32,33,34,35]. The authors suggested this difference is due in part to how owners of small dogs interact with their animals, with owners of small dogs showing more inconsistency in their interactions, and reporting lower levels of training, exercise and human-dog play behaviour [34,35,36]. Poor behaviour from smaller dogs is also tolerated better by the public, as demonstrated by statistics on dog bite occurrence [34,35]. On average, smaller breed dogs bite more frequently and leave lacerations on their human bite victims, while larger breed dogs do not bite as often as smaller dogs but create more damage [34]. Because of this, larger dogs that have broken skin are much more likely to be euthanized due to aggressive behaviour than smaller dogs that display the same behaviour but create less damage [34]. Owner lifestyle, demographics, ethnicity and living location may also play a role in how small dog owners interact with their dog [34]. Because of this tolerance towards poor behaviour in smaller dogs, it would not be surprising that less selection against poor behaviour may occur in small breed dogs [34,35]. There is also evidence that suggests body size, weight and skull shape may have an impact on different behaviours which may provide some explanation for the variation observed between the large and small purebreds included in the current study [35]. Further research is needed to determine whether the type of Poodle that is used in the cross-influences the behaviour of Doodle breeds.

As with all research, this study has certain limitations that might have influenced the findings and conclusions. There may be some breed identification errors in the data for the dogs included in the current study; C-BARQ records are based on owner reports, thus, both purebreds and cross-breeds might be misclassified by owners. For the same reason, we also do not know how related the dogs in the study are or if they are from the same breeder or origin. Owner information was accounted for, and we have assumed no relationship between dogs and that all crosses are F1 crosses. In addition, we had a relatively small sample size of crossbred dogs when compared to our purebred animals, which may impact the generalisability of our results, in spite of the Tukey-Kramer adjustment used to account for unbalanced data.

## 5. Conclusions

As expected from an F1 cross, the cross-breeds tended to express intermediate behaviour between the two constituent pure-breeds with some exceptions. The GD analysis showed the most difference between the constituent pure-breeds. Even with these differences, the Goldendoodle only differed significantly from one or more of the parent-breeds in dog-directed aggression, dog-directed fear, stranger-directed fear, and dog rivalry. The crossbred in the LD analysis showed the fewest behavioural differences from the other parent-breeds with the exception of dog rivalry, but that was due to the Miniature Poodle differing from the other breeds in the LD analysis. These results suggest that differential expression of the retriever-based behavioural tendencies is dependent on the retriever origin. Further research is needed to determine whether *IGF1* expression might play a role in behavioural tendencies for poodle crosses, and how this is influenced by contributions from the non-poodle parent. Overall, the results of the current study can assist breeders and owners in predicting likely behavioural phenotypes for LD and GD crossbreds, and this information can be used to assist owners in selecting breeds that best fit their needs and expectations.

## Figures and Tables

**Table 1:** Mean owner-reported Canine Behavioural Assessment and Research Questionnaire (C-BARQ) scores for main behavioural categories for Labrador Retrievers (n = 2597), Labradoodles (n = 166), Standard Poodles (n = 597) and Miniature Poodles (n = 258). Model interactions between breed and other fixed effects are also reported. Breed comparison indicates when breeds differed.

Trait	Model Effect					Breed Mean Behavioural Scores			
	Breed	Breed × Sex	Breed × Country	Breed × Where Acquired	Breed × Age at Evaluation	Labrador Retriever	Labradoodle	Miniature Poodle	Standard Poodle
Trainability		●	●	●	●	2.63	2.73	2.54	2.70
Stranger aggression		●	●	●	●	0.47	0.56	0.65	0.57
Owner aggression	●	●	●	●	●	0.23	0.12	0.32	0.16
Dog aggression		●	●	●	●	0.95	0.62	0.92	0.74
**Dog Rivalry**	●		●	●	●	**0.44 ^a^**	**0.19 ^a^**	**1.02 ^b^**	**0.37 ^a^**
Dog fear			●	●	●	0.74	0.90	0.78	0.73
Stranger Fear			●	●		0.50	0.69	0.65	0.66
**Non-Social fear**	●		●	●		**0.68 ^a^**	**0.91 ^ab^**	**1.04 ^b^**	**0.72 ^ab^**
**Touch sensitivity**	●		●	●		**0.72 ^ab^**	**0.76 ^ab^**	**1.04 ^a^**	**0.59 ^b^**
**Separation-related problems**	●	●	●	●	●	**0.55 ^a^**	**0.76 ^ab^**	**0.93 ^b^**	**0.62 ^ab^**
Excitability			●	●		2.00	2.04	2.28	1.93
Attachment/attention-seeking behaviours		●	●	●	●	2.04	2.16	2.03	1.98
Chasing				●	●	1.84	1.65	1.86	1.97
Energy		●		●	●	2.11	2.20	2.04	2.07

● Significant difference for the indicated variable or interaction (*p* < 0.05). ^a,b^ Different letters within the same row denote significant differences (*p* < 0.05*).* Bolded text indicate traits that displayed significant differences between breeds.

**Table 2:** Mean owner-reported C-BARQ scores for main behavioural categories for Golden Retrievers (n = 1366), Goldendoodles (n = 157), Standard Poodles (n = 597) and Miniature Poodles (n =258. Model interactions between breed and other fixed effects are also reported. Breed comparison indicates when breeds differed.

Trait	Model Effect					Breed Mean Behavioural Scores			
	Breed	Breed × Sex	Breed × Country	Breed × Where Acquired	Breed × Age at Evaluation Weeks	Golden Retriever	Goldendoodle	Miniature Poodle	Standard Poodle
Trainability			●	●		2.61	2.91	2.54	2.69
Stranger aggression		●	●	●	●	0.56	0.85	0.67	0.56
**Owner aggression**	●	●	●	●	●	**0.35 ^a^**	**0.19 ^ab^**	**0.32 ^ab^**	**0.17 ^b^**
**Dog aggression**	●	●	●	●	●	**0.73 ^a^**	**1.68 ^b^**	**0.92 ^a^**	**0.74 ^a^**
**Dog Rivalry**	●		●	●	●	**0.42 ^a^**	**0.37 ^a^**	**1.05 ^b^**	**0.38 ^a^**
**Dog fear**	●		●		●	**0.82 ^ab^**	**1.44 ^a^**	**0.79 ^ab^**	**0.74 ^b^**
**Stranger Fear**	●	●	●	●	●	**0.48 ^a^**	**1.16 ^b^**	**0.65 ^ab^**	**0.66 ^ab^**
Non-Social fear			●	●	●	0.93	1.12	1.04	0.73
**Touch sensitivity**	●		●	●	●	**0.65 ^a^**	**0.75 ^ab^**	**1.04 ^b^**	**0.61 a**
**Separation-related problems**	●	●	●	●	●	**0.51 ^a^**	**0.93 ^ab^**	**0.94 ^b^**	**0.63 ^ab^**
Excitability				●	●	1.97	2.21	2.29	1.94
Attachment/attention-seeking behaviours		●	●			1.89	2.02	2.01	1.98
Chasing			●		●	1.74	2.23	1.84	1.98
Energy				●	●	2.01	2.05	2.05	2.04

● Significant difference for the indicated variable or interaction (*p* < 0.05). ^a,b^ Different letters within the same row denote significant differences (*p* < 0.05). Bolded text indicate traits that displayed significant differences between breeds.
